# Correction: Integration of evidence into Theory of Change frameworks in the healthcare sector: A rapid systematic review

**DOI:** 10.1371/journal.pone.0318028

**Published:** 2025-01-17

**Authors:** The PLOS ONE Staff

The captions for Figs 1–3 are incorrect. The correct captions have been provided here:

**Fig 1:**
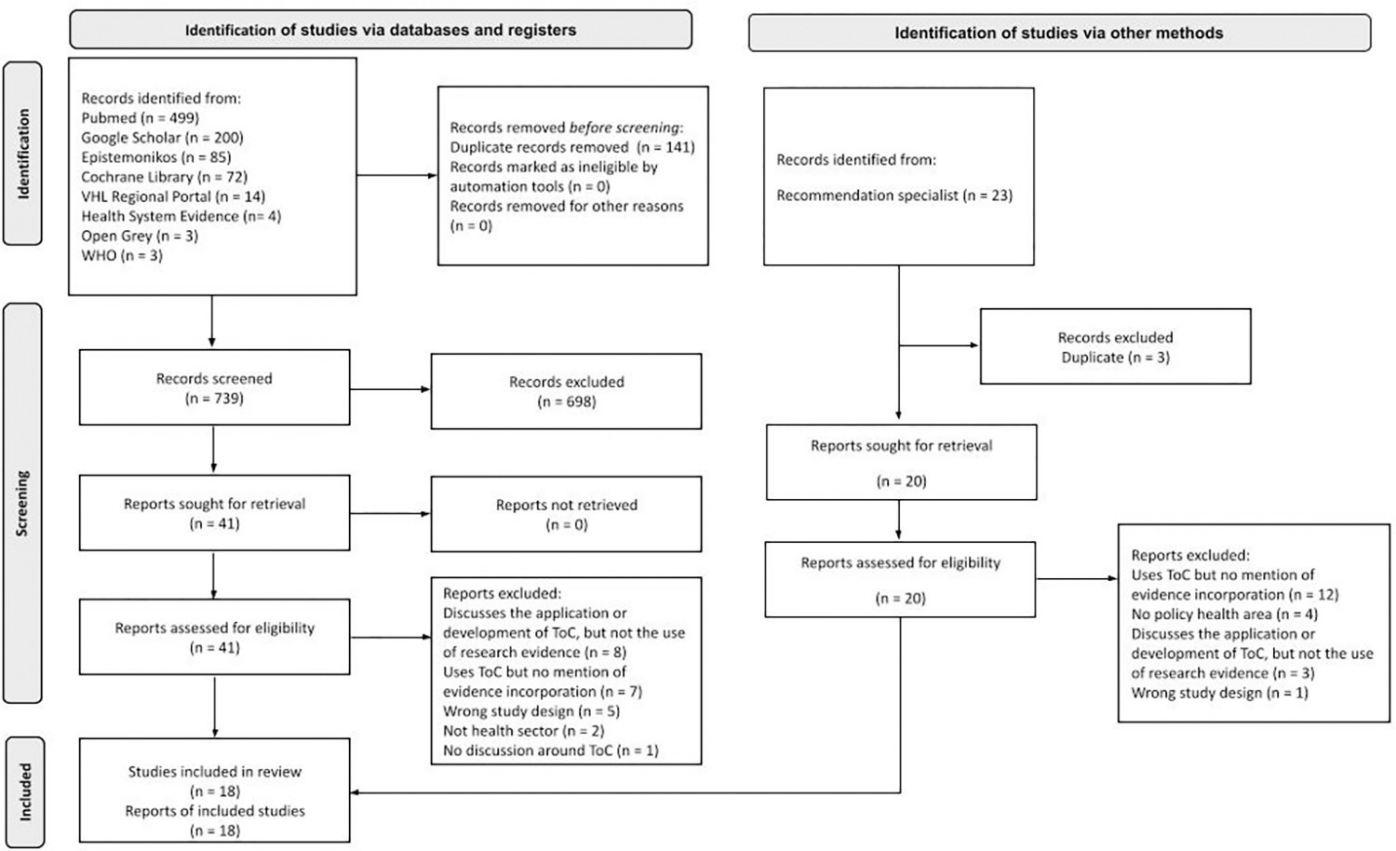
PRISMA flowchart.

Source: authors’ elaboration, adapted from PRISMA 2020 [35].

**Fig 2:**
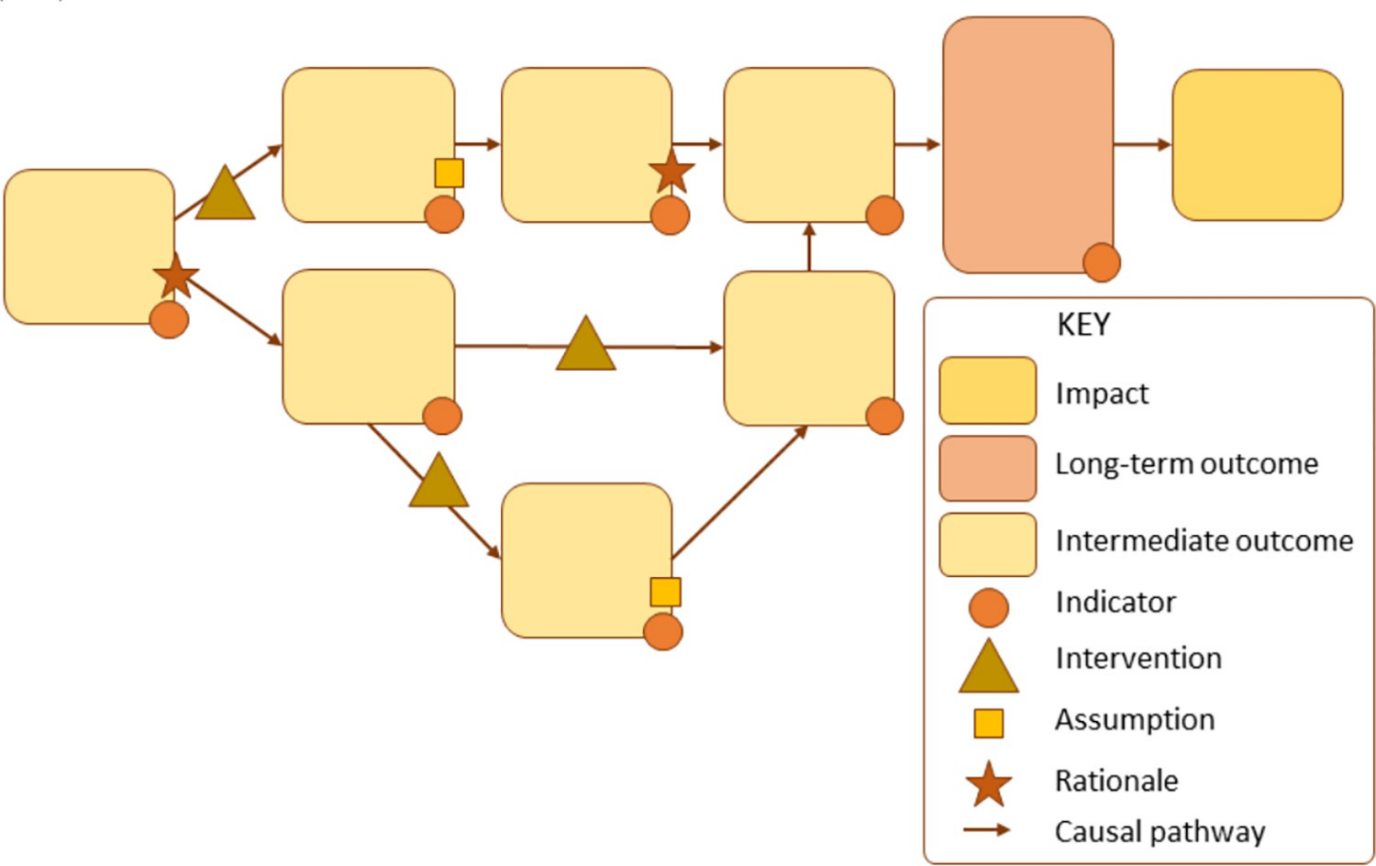
Example of Theory of Change framework.

Source: De Silva et al., 2014, p.4 [13].

**Fig 3:**
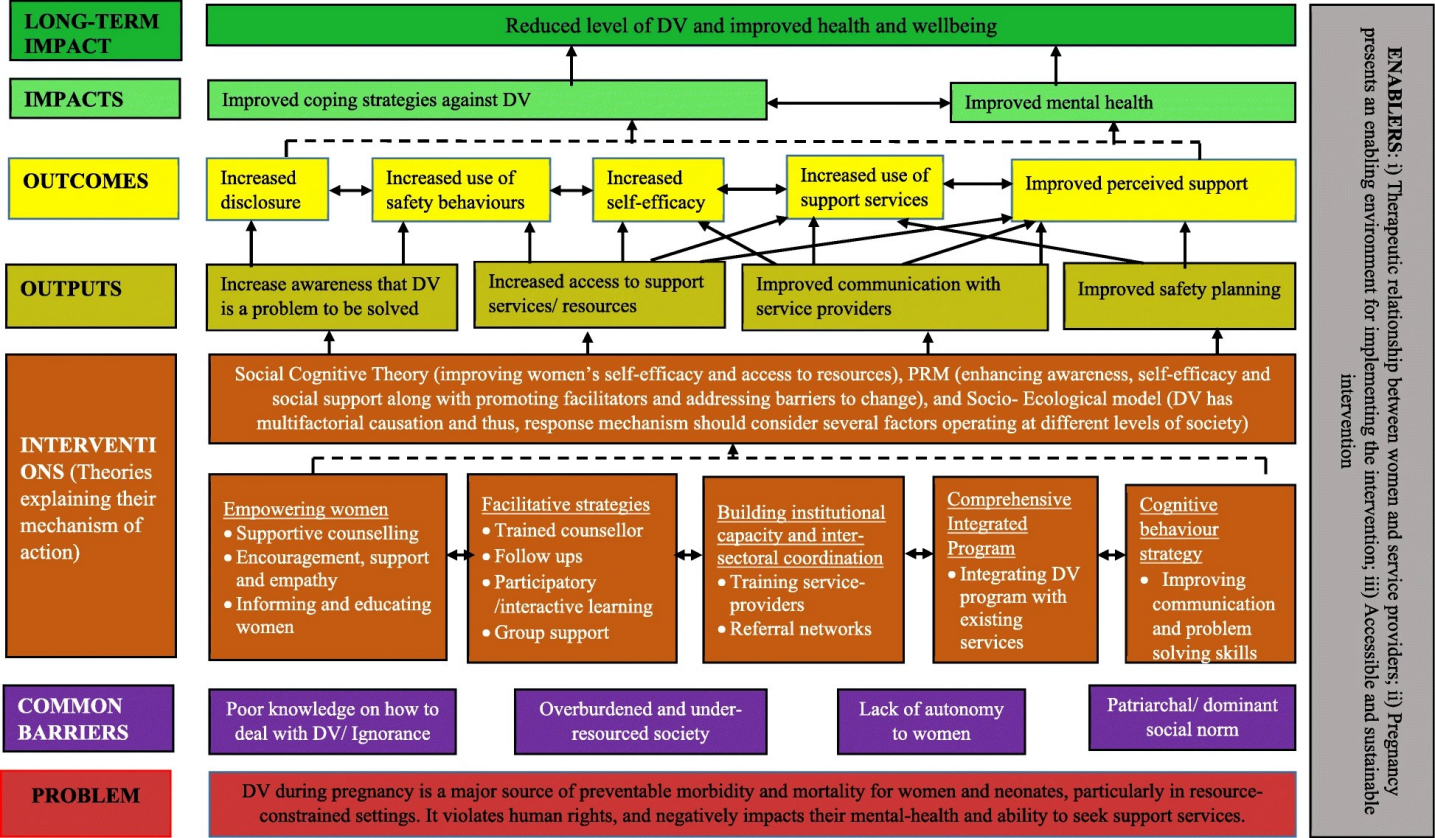
Example of theory of change for an intervention.

Source: Sapkota et al., 2019, p.6 [14].

The publisher apologizes for the error.
