# Supplementary material for: Integration of evidence into Theory of Change frameworks in the healthcare sector: A rapid systematic review
Source: PLoS One. 2023 Mar 9;18(3):e0282808. doi: 10.1371/journal.pone.0282808 (PMC9997872; doi:10.1371/journal.pone.0282808)
Supplement: S1 Appendix — (DOCX) [file pone.0282808.s001.docx]

### S1 Appendix. Search strategies

| **Base** | **Date** | **Query** | **Results** |
| --- | --- | --- | --- |
| PubMed | 08/11/2021 | "Theory of Change" | 499 |
| BVS | 08/11/2021 | "theory of change" AND ( db:("LILACS" OR "IBECS" OR "INDEXPSI" OR "PAHOIRIS")) | 14 |
| Cochrane Library | 08/11/2021 | ID Search Hits  #1 "Theory of Change" 81 | 72 |
| HSE | 08/11/2021 | ("Theory of Change") | 4 |
| Epistemonikos | 08/11/2021 | ("Theory of Change") | 85 |
| OpenGray | 08/11/2021 | ("Theory of Change") AND Health | 3 |
| Google Scholar | 08/11/2021 | ("Theory of Change") AND Health | 200 |
| WHO | 08/11/2021 | "Theory of Change" | 3 |
| **Total** | | | **880** |

### Source: authors' elaboration.
