# Supplementary material for: Integration of evidence into Theory of Change frameworks in the healthcare sector: A rapid systematic review
Source: PLoS One. 2023 Mar 9;18(3):e0282808. doi: 10.1371/journal.pone.0282808 (PMC9997872; doi:10.1371/journal.pone.0282808)
Supplement: S2 Appendix — *Critical domains; CL: Critically low. (DOCX) [file pone.0282808.s002.docx]

### S2 Appendix. Methodological quality of the included studies

**S2A Figure. Methodological quality of the systematic reviews assessed by AMSTAR 2.** Source: authors' elaboration.*Critical domains; CL: Critically low.

**S2A Table. JBI Critical Appraisal Checklist for systematic reviews and research syntheses**

| **Lead Author/Year** | **1. Is the review question clearly and explicitly stated?** | **2. Were the inclusion criteria appropriate for the review question?** | **3. Was the search strategy appropriate?** | **4. Were the sources and resources used to search for studies adequate?** | **5. Were the criteria for appraising studies appropriate?** | **6. Was critical appraisal conducted by two or more reviewers independently?** | **7. Were there methods to minimize errors in data**  **extraction?** | **8. Were the methods used to combine studies appropriate?** | **9. Was the likelihood of publication bias assessed?** | **10. Were recommendations for policy and/or practice supported by the reported data?** | **11. Were the specific directives for new research appropriate?** |
| --- | --- | --- | --- | --- | --- | --- | --- | --- | --- | --- | --- |
| Lam et al. 2021[24] | Yes | Yes | Yes | Yes | Not applicable | Not applicable | No | Yes | Not applicable | Yes | Yes |

Source: authors' elaboration.

**S2B Table. JBI Critical appraisal checklist for qualitative research**

| **Lead Author/year** | **1. Is there congruity between the stated philosophical perspective and the research methodology?** | **2. Is there congruity between the research methodology and the research question or objectives?** | **3. Is there congruity between the research methodology and the methods used to collect data?** | **4. Is there congruity between the research methodology and the representation and analysis of data?** | **5. Is there congruity between the research methodology and the interpretation of results?** | **6. Is there a statement locating the researcher culturally or theoretically?** | **7. Is the influence of the researcher on the research, and vice- versa, addressed?** | **8. Are participants, and their voices, adequately represented?** | **9. Is the research ethical according to current criteria or, for recent studies, and is there evidence of ethical approval by an appropriate body?** | **10. Do the conclusions drawn in the research report flow from the analysis, or interpretation, of the data?** |
| --- | --- | --- | --- | --- | --- | --- | --- | --- | --- | --- |
| Aromatario et al. 2019 [20] | Yes | Yes | Yes | Not applicable | Yes | No | Not applicable | Not applicable | No | Yes |
| De Buck et al. 2018 [1] | Yes | Yes | Yes | Yes | Yes | Yes | Not applicable | Not applicable | No | Yes |
| Hartley et al. 2019 [36] | Yes | Yes | Yes | Not applicable | Yes | No | No | Yes | Yes | Yes |

| **Lead Author/year** | **1. Is there congruity between the stated philosophical perspective and the research methodology?** | **2. Is there congruity between the research methodology and the research question or objectives?** | **3. Is there congruity between the research methodology and the methods used to collect data?** | **4. Is there congruity between the research methodology and the representation and analysis of data?** | **5. Is there congruity between the research methodology and the interpretation of results?** | **6. Is there a statement locating the researcher culturally or theoretically?** | **7. Is the influence of the researcher on the research, and vice- versa, addressed?** | **8. Are participants, and their voices, adequately represented?** | **9. Is the research ethical according to current criteria or, for recent studies, and is there evidence of ethical approval by an appropriate body?** | **10. Do the conclusions drawn in the research report flow from the analysis, or interpretation, of the data?** |
| --- | --- | --- | --- | --- | --- | --- | --- | --- | --- | --- |

| Jamal et al. 2015 [37] | Yes | Yes | Yes | Not applicable | Not applicable | No | Not applicable | Not applicable | Yes | Yes |
| --- | --- | --- | --- | --- | --- | --- | --- | --- | --- | --- |
| Tirman et al. 2021 [42] | Yes | Yes | Yes | Yes | Yes | No | No | Yes | Yes | Yes |
| Yearwood 2018[43] | Yes | Yes | Yes | Yes | Yes | No | No | Yes | No | Yes |

Source: authors' elaboration.

**S2C Table. JBI Critical Appraisal Checklist for text and opinion papers**

| **Lead Author/Year** | **1. Is the source of the opinion clearly identified?** | **2. Does the source of opinion have standing in the field of expertise?** | **3. Are the interests of the relevant population the central focus of the opinion?** | **4. Is the stated position the result of an analytical process, and is there logic in the opinion expressed?** | **5. Is there reference to the extant literature?** | **6. Is any incongruence with the literature/sources logically defended?** |
| --- | --- | --- | --- | --- | --- | --- |
| De Silva et al. 2014[13] | Yes | Yes | Not applicable | Not applicable | Yes | Yes |
| Rippon S et al., 2017[40] | Yes | Yes | Not applicable | Not applicable | Yes | Yes |

Source: authors' elaboration.

**S2D Table. Scale for the Assessment of Narrative Review Articles (SANRA)**

| **Lead Author/Year** | **1.Justification of the article's importance for the readership** | **2.Statement of concrete aims or formulation of questions** | **3. Description of the literature search** | **4. Referencing** | **5. Scientific reasoning** | **6. Appropriate presentation of data** | **Total** |
| --- | --- | --- | --- | --- | --- | --- | --- |
| Mayne, Johnson 2015[38] | 2- The importance is explicitly justified | 2-One or more concrete aims or questions are formulated | 1-The literature search is described briefly | 2-Key statements are supported by references | 1- Appropriate evidence is introduced selectively | 2- Relevant outcome data are generally presented appropriately | 10/12 |

Source: authors' elaboration.
