# Supplementary material for: Integration of evidence into Theory of Change frameworks in the healthcare sector: A rapid systematic review
Source: PLoS One. 2023 Mar 9;18(3):e0282808. doi: 10.1371/journal.pone.0282808 (PMC9997872; doi:10.1371/journal.pone.0282808)
Supplement: S4 Appendix — (DOCX) [file pone.0282808.s004.docx]

### S4 Appendix. Full-text articles excluded, with reasons

| **References retrieved from databases** | |
| --- | --- |
| **Reason: Discusses the application or development of ToC, but not the use of research evidence** | |
| 1 | Bonell C, Hinds K, Dickson K, Thomas J, Fletcher A, Murphy S, et al. What is positive youth development and how might it reduce substance use and violence? A systematic review and synthesis of theoretical literature. BMC Public Health. 2016;16:135. |
| 2 | Breuer E, De Silva MJ, Shidaye R, Petersen I, Nakku J, Jordans MJ, et al. Planning and evaluating mental health services in low- and middle-income countries using theory of change. Br J Psychiatry. 2016;208:s55-62. |
| 3 | Douthwaite B, Kamp K, Longley C, Kruijssen F, Puskur R, Chiuta T, et al. Using theory of change to achieve impact in AAS. 2013. |
| 4 | Fuhr DC, Acarturk C, Sijbrandij M, Brown FL, Jordans MJD, Woodward A, et al. Planning the scale up of brief psychological interventions using theory of change. BMC Health Serv Res. 2020;20(1):801. |
| 5 | Fuhr DC, Acarturk C, Uygun E, McGrath M, Ilkkursun Z, Kaykha S, et al. Pathways towards scaling up Problem Management Plus in Turkey: a theory of change workshop. Confl Health. 2020;14:22. |
| 6 | Segal L, Sara Opie R, Dalziel K. Theory! The missing link in understanding the performance of neonate/infant home-visiting programs to prevent child maltreatment: a systematic review. Milbank Q. 2012;90(1):47–106. |
| 7 | Swendeman D, Ramanathan N, Baetscher L, Medich M, Scheffler A, Comulada WS, et al. Smartphone self-monitoring to support self-management among people living with HIV: perceived benefits and theory of change from a mixed-methods randomized pilot study. J Acquir Immune Defic Syndr. 2015;69:S80-91. |
| 8 | Baffsky R, Kemp L, Bunde-Birouste A. Theory of Change in Sports-Based Urban Youth Programs: Lessons from Creating Chances. In: Oxford Research Encyclopedia of Global Public Health. 2021. |
| **Reason: Uses ToC but no mention of evidence incorporation** | |
| 9 | Baker C, Courtney P, Knepil G. Evaluating societal outcomes of orthognathic surgery: an innovative application of the Social Return on Investment methodology to patients after orthognathic treatment. Br J Oral Maxillofac Surg. 2019;57(2):145–50. |
| 10 | Baraitser P, Syred J, Spencer-Hughes V, Howroyd C, Free C, Holdsworth G. How online sexual health services could work; generating theory to support development. BMC Health Serv Res. 2015;15:540. |
| 11 | Jeukens-Visser M, Koldewijn K, van Wassenaer-Leemhuis AG, Flierman M, Nollet F, Wolf MJ. Development and nationwide implementation of a postdischarge responsive parenting intervention program for very preterm born children: The TOP program. Infant Ment Health J. 2021;42(3):423–37. |
| 12 | King J. Expanding theory-based evaluation: Incorporating value creation in a theory of change. Eval Program Plann. 2021;89:101963. |
| 13 | King KM, Littlefield AK, McCabe CJ, Mills KL, Flournoy J, Chassin L. Longitudinal modeling in developmental neuroimaging research: Common challenges, and solutions from developmental psychology. Dev Cogn Neurosci. 2018;33:54–72. |
| 14 | Maureen Seguin, Miguel Niño-Zarazúa. What Do We Know About Non-clinical Interventions for Preventable and Treatable Childhood Diseases in Developing Countries? [Internet]. 2013. (International Initiative for Impact Evaluation (3ie)). Disponível em: http://www.epistemonikos.org/documents/554aa3cd82de2d1bbdca01b6bb40a850087cdd94 |
| 15 | O’Hara JK, Baxter R, Hardicre N. “Handing over to the patient”: A FRAM analysis of transitional care combining multiple stakeholder perspectives. Appl Ergon. 2020;85:103060. |
| **Reason: Wrong study design** | |
| 16 | Breuer E, DeSilva M, Shidaye R, Petersen I, Fekadu A, Nakku J, et al. Using theory of change as an approach to design complex mental health interventions: lessons from PRIME. |
| 17 | Chaskin RJ. Toward a theory of change in community-based practice with youth: A case-study exploration. Children and Youth Services Review. 2009;31(10):1127–34. |
| 18 | Giersing B, Shah N, Kristensen D, Amorij JP, Kahn AL, Gandrup-Marino K, et al. Strategies for vaccine-product innovation: Creating an enabling environment for product development to uptake in low- and middle-income countries. Vaccine [Internet]. 2021 [cited Jan 1srt 10DC]; Disponível em:<https://pubmed.ncbi.nlm.nih.gov/34627624/> |
| 19 | Gultekin L, Kusunoki Y, Sinko L, Cannon L, Abramoski K, Khan AG, et al. The Eco-Social Trauma Intervention Model. Public Health Nurs. 2019;36(5):709–15. |
| 20 | Lederer AM, Johnson KM, Liddell JL, Sheffield S. The Multimethod Evaluation of a Curricular Intervention Intended to Reduce Sexual Violence on a College Campus: A Synthesis of Findings and Lessons Learned. Health Promot Pract. 2021;15248399211050348. |
| **Reason: Not from health sector** | |
| 21 | Biggs D, Cooney R, Roe D, Dublin HT, Allan JR, Challender DW, et al. Developing a theory of change for a community-based response to illegal wildlife trade. Conserv Biol. 2017;31(1):5–12 |
| 22 | Weitzman BC, Silver D, Dillman K-N. Integrating a comparison group design into a theory of change evaluation: The case of the Urban Health Initiative. American Journal of Evaluation. 2002;23(4):371–85. |
| **Reason: No discussion around ToC** | |
| 23 | Birnbaum ML, Daily EK, O’Rourke AP, Kushner J. Research and Evaluations of the Health Aspects of Disasters, Part VI: Interventional Research and the Disaster Logic Model. Prehosp Disaster Med. 2016;31(2):181–94. |
| **References recommended by specialist** | |
| **Reason: Uses ToC but no mention of evidence incorporation** | |
| 1 | Funnell SC, Rogers PJ. **Purposeful program theory: Effective use of theories of change and logic models**. John Wiley & Sons, 2011. |
| 2 | Guijt I. ToC reflection notes 3: Working with assumptions in a theory of change process. 2013. |
| 3 | James C. Theory of change review. **Comic Relief**, 2011. |
| 4 | Pasanen T, Barnett I. Supporting adaptive management: monitoring and evaluation tools and approaches. 2019. |
| 5 | Stronger Collaboration, Better Health – Global Action Plan for Healthy Lives and Well-being for All. Theory of change. November, 2020. |
| 6 | Taplin DH, Clark H, Collins E, Colby DC. Theory of change. *Technical papers: a series of papers to support development of theories of change based on practice in the field. ActKnowledge, New York, NY, USA*, 2013. |
| 7 | United Nations Environment Programme. Monitoring, Evaluation and Learning Strategy and Action Plan Glossary. 2020 |
| 8 | UK Aid Connect: Guidance Note: Developing a Theory of Change [s.d.] |
| 9 | Van Es M, Guijt I, Vogel I. Hivos ToC GuidelinesTheory of Change Thinking in Practice. 2015. |
| 10 | Valters C. Theories of Change: Time for a Radical Approach to Learning in Development (Overseas Development Institute). 2015. |
| 11 | Vogel I. ESPA guide to working with Theory of Change for research projects. **Ecosystem Services for Alleviation of Poverty,** 2012. |
| 12 | Vogel I. Review of the use of ‘Theory of Change’ in international development Review Report. August 2012. |
| **Reason: Not from health sector** | |
| 13 | Anderson AA. The community builder’s approach to Theory of Change. 2006 |
| 14 | Mayne J. Theory of Change Analysis: Building Robust Theories of Change. Canadian Journal of Program Evaluation. 2017 Dec 4;32. |
| 15 | Rogers P. *Theory of change: methodological briefs-impact evaluation No. 2*. No. Innpub747. 2014. |
| 16 | UNSDG \| THEORY OF CHANGE UNDAF COMPANION GUIDANCE [Internet]. [cited 2021 Dec 7]. Available from: [https://unsdg.un.org/resources/theory-change-undaf-companion-guidance, https://unsdg.un.org/resources/theory-change-undaf-companion-guidance](https://unsdg.un.org/resources/theory-change-undaf-companion-guidance,%20https:/unsdg.un.org/resources/theory-change-undaf-companion-guidance) |
| **Reason: Discusses the application or development of ToC, but not the use of research evidence** | |
| 17 | De Silva M, Lee L, Ryan G. Using Theory of Change in the development, implementation and evaluation of complex health interventions. **London: The Centre for Global Mental Health at the London School of Hygiene & Tropical Medicine and The Mental Health Innovation Network**, 2014. |
| 18 | Stein D, Valters C. Understanding theory of change in international development. JSRP, 2012. |
| 19 | UNICEF. Theory of Change UNICEF Strategic Plan, 2022–2025 (SRS 2021) [Internet]. [cited 2021 Dec 7]. Available from: <https://www.unicef.org/executiveboard/documents/theory-change-paper-unicef-strategic-plan-2022-2025-SRS-2021> |
| **Reason: Wrong study design** | |
| 20 | Vogel I. Theory of change and research impact. ESPA, 2012. |

Source: authors' elaboration.
