# Supplementary material for: Integration of evidence into Theory of Change frameworks in the healthcare sector: A rapid systematic review
Source: PLoS One. 2023 Mar 9;18(3):e0282808. doi: 10.1371/journal.pone.0282808 (PMC9997872; doi:10.1371/journal.pone.0282808)
Supplement: S5 Appendix — *Study type: When the authors did not inform the study design, the reviewers attributed a classification based on the provided description of the methods. HIC: High-income economies; LMIC: Lower-middle-income economies; UMIC: Upper-middle-income economies. ** The systematic review had a ToC as one of its outputs. (DOCX) [file pone.0282808.s005.docx]

**S5 Appendix. Characteristics of the included studies**

| **Lead Author/Year** | ***Study type** | **Objective** | **Total of primary studies focusing on ToC** | **Countries of studies about ToC** | **Participants in the ToC development** |
| --- | --- | --- | --- | --- | --- |
| Aggarwal et al. 2021 [19] | Systematic Review | To synthesise the research literature on the implementation and effectiveness of interventions for reducing self-harm in low- and middle-income countries. The specific objectives of the current review are:  - To conduct a systematic review of studies on the implementation, adaptation or effectiveness of psychosocial interventions for self-harm in LMICs.  - To identify effective components and the delivery mechanisms of these interventions, using a narrative synthesis. To create a ToC map from an evaluation of interventions, indicators, assumptions, rationales, outcomes as well as contextual and motivational factors to guide delivery of future interventions in LMIC contexts. | 13 studies | HIC: Australia, USA  LMIC: India | Not reported |
| Aromatario et al. 2019 [20] | Not reported (methodological study) | The aim of the OCAPREV project (Objets Connectéset Application en PREVention - Connected devices and applications for prevention) is to elaborate an intervention theory for SDApps that support healthy eating and physical activity for adults over 18, with the emphasis on social health inequalities (SHI). This article sets out how the theory was elaborated using the ToC model, and presents the theory as a potential framework for designing and evaluating behavior change SDApps in the two above-mentioned areas. | Not applicable | Not reported | - Multidisciplinary professional group: researchers with various backgrounds including learning sciences, sports sciences, public health, psychology and sociology; healthcare and prevention practitioners; professionals from fields relating to healthy eating and physical activity, with a general practitioner, a private dietician, and two health educators including a psychologist; a user representative; and a smartphone app designer (private firm).  - User group was composed of 12 individuals, 5 men and 7 women above the age of 18, who were interested and who volunteered. They were included because they were part of a cardiovascular disease prevention network. |
| Barnhart et al. 2020 [18] | Case study | Using data from the BetterBirth Program and its associated trial as a case study, we identified lessons to assist in the development and evaluation of future complex interventions | Not applicable | HI: USA  LMIC: India | Program evaluators and implementers, community members |
| Bonell et al. 2013 [21] | Systematic review | To explain or hypothesise how the school environment influences health. The objective of this synthesis was to construct an integrated theory delineating the complex causal pathways via which school effects on health may occur, which might inform future interventions and research. | 0 studies** | HIC: UK | Not reported |
| Breuer et al. 2016 [23] | Systematic review | To determine how ToCs have been developed and used in the development and evaluation of public health interventions globally. Specifically, we sought to answer the following questions:  (1) How are ToCs for public health interventions developed and refined?  (2) How is the ToC approach used in the  (a) development of an intervention;  (b) implementation of the intervention;  (c) development of indicators for measurement;  (d) evaluation of the intervention, including statistical approaches; and  (e) conceptualisation/evaluation of the influence of context. | 62 studies | HIC: UK, USA and other countries | Not reported |
| De Buck et al. 2018 [1] | Not reported (qualitative study) | To describe the development of a ToC that is relevant for practice. | Not applicable | Not reported | Methodological experts (systematic review, qualitative research) and a topical expert in WASH behaviour change. |
| De Silva et al. 2014 [13] | Formative research | This guide provides a practical overview of the process of developing a Theory of Change, focussing on using a stakeholder-driven, workshop approach to achieve this. | Not applicable | Not reported | Not reported |
| Hartley et al. 2019 [36] | Methodological study | To apply to kinship care in Scotland an intervention development framework derived from the Six Steps in Quality Intervention Development (6SQuID). | Not applicable | HIC: Scotland, UK | Authors (based on semi-structured interviews with kinship carers and discussions with advisory group) |
| Jamal et al. 2015 [37] | Methodological study | This paper provides a worked example of the theoretical and methodological process of undertaking a realist RCT drawing on the evaluation of the Learning Together (LT) intervention. | Not applicable | HIC: England, UK | Not reported |
| Lam et al. 2021 [24] | Scoping review | To examine the nature, extent, and range of published and grey literature on ToCs and REs used in the context of food security;  To examine the assumptions, conditions, and problem framings that allow ToCs and REs to emerge as appropriate tools to guide the evaluation of food security programs. In doing so, we become better equipped to make methodological choices that align with the evaluation setting and the attributes of the food security program. | 59 studies | África (41 studies)  Asia (14 studies)  North America (4 studies)  South America (2 studies)  Europe (2 studies)  Australia and Oceania (2 studies)  Study did not specify characteristics of case studies (e.g. conceptual/theo- retical papers) | Not reported |
| Mayne; Johnson, 2015 [38] | Not reported (methodological study) | In this article, we first provide a brief introduction to IPs and ToCs and their use in the context of A4NH research activities. We then set out the generic components of IPs and ToCs for the kinds of complex interventions the A4NH research program is involved with. We then discuss the various ways that ToCs can be used in planning, managing and assessing A4NH interventions. It concludes by discussing key characteristics of A4NH ToCs, most of which apply to other multifaceted interventions. | Not applicable | Not reported | Not reported |
| Meiksin et al. 2021 [22] | Systematic review | To explore the approaches and theories of how the interventions were intended to work to improve targeted health outcomes(“theories of change”) employed; factors promoting or impeding delivery or receipt of such interventions; their effectiveness in improving health outcomes and whether such interventions are cost-effective. | 37 studies | Not reported | Authors |
| Osterman et al. 2021 [39] | Realist systematic review | This study synthesized evidence of effective interventions for improving data use in decision-making. | 6 studies | Not reported | Review team |
| Rippon et al. 2017 [40] | Report | To explore two key areas that we see as being critical for moving to a more systematised approach to asset based action for health. These two areas are:1. The need to develop further a Theory of Change for asset based approaches aligned to an asset model for health2. The requirement to understand how to measure and illustrate impact and benefit from asset based approaches. | Not applicable | Not reported | Authors |
| Sapkota et al. 2019 [14] | Systematic review  Included | To obtain a complete representation of interventions available in Low- and middle-income countries (LMICs) for reducing and/or controlling Domestic violence (DV) among pregnant women and assess their effectiveness. | 5 studies | LMIC (Not reported) | Not reported |
| Tancred et al. 2018 [41] | Systematic review | As a first step, and within the context of a broader systematic review also synthesising evidence from process and outcome evaluations of school-based interventions that integrate health and academic education to reduce violence or substance use among children and young people, we used meta-ethnographic methods to synthesise theories of change for such interventions. | 9 studies | Not reported | Not reported |
| Tirman et al. 2021 [42] | Mixture of qualitative and quantitative methods | To develop a theory-based sedentary behaviour intervention for contact centre workers, known as Stand Up for Health (SUH), using the 6SQuID framework.  The objectives were to:  1 Define and understand sedentary behaviour in the specific contact centre (6SQuID Steps 1 & 2);  2 Identify the non-modifiable and modifiable causal factors of sedentary behaviour in the contact centre (6SQuID Step 2);  3 Develop intervention activities and identify resources needed to target the modifiable causal factors (6SQuID Steps 3 & 4);  4 Introduce the activities with contact centre staff while refining the programme theory (Step 5). | Not applicable | HIC: Scotland | Staff members Ipsos MORI contact centre |
| Yearwood, 2018 [43] | Not reported (methodological study) | To develop a ToC to narrow the research-policy gap in the Caribbean;  To assess its design logic; and iii) to evaluate the extent to which the main expected outcome was achieved. | Not applicable | UMIC: the Caribbean | Not reported |

Source: authors' elaboration. *Study type: When the authors did not inform the study design, the reviewers attributed a classification based on the provided description of the methods. HIC: High-income economies; LMIC: Lower-middle-income economies; UMIC: Upper-middle-income economies. ** The systematic review had a ToC as one of its outputs.
