# Supplementary material for: Integration of evidence into Theory of Change frameworks in the healthcare sector: A rapid systematic review
Source: PLoS One. 2023 Mar 9;18(3):e0282808. doi: 10.1371/journal.pone.0282808 (PMC9997872; doi:10.1371/journal.pone.0282808)
Supplement: S6 Appendix — (DOCX) [file pone.0282808.s006.docx]

### S6 Appendix. Results of the included studies

| **Lead Author/Year** | **Results** | | | |
| --- | --- | --- | --- | --- |
|  | **Use of research evidence in the development of a ToC** | **Use of research evidence for revising a ToC** | **Steps for the development of a ToC** | **Steps for revising a Theory of Change** |
| Aggarwal et al. 2021 [19] | The ToC presented by the authors was based on the findings of the Systematic Review carried out and presented in this same article | Not Reported | The ToC was first conceptualised and drafted by the lead reviewer (SA), drawing on the findings of the studies. A senior reviewer with expertise in designing complex interventions using ToC model evaluated the ToC map, and a working draft was agreed between the two reviewers. The map was finalised in consensus with a third reviewer. [...]  Drawing upon the findings of the studies included in the review, was proposed a model of delivery of intervention based on theory of change (ToC)[...].  We used conceptual mapping to identify themes from the studies that could be used for the ToC model [38].  The ToC map shows the multiple causal pathways through which the outcomes and activities work to achieve the desired impact.  The findings from the review studies as well as past evidence have informed the rationale for the interventions needed to move from one precondition on the causal pathway to the next.  We identified the gaps in the research, the potential barriers and the interventions needed to overcome these barriers along the way.  Furthermore, indicators for the achievement of each precondition in the pathway is listed to evaluate whether every stage of the pathway leads to the final impact. | Not Reported |
| Aromatario et al. 2019 [20] | Step 1 - identifying the evidence base. This step aims to identify and select data from the literature that could help stakeholders involved in phase 2 in designing the intervention theory.  Step 2 - developing the theory. In keeping with theory-driven approaches, we put together 4 expert focus groups and one user focus group in order to elaborate the intervention theory. An e-Delphi method was then used to enable all the expert panellists to reach a consensus by validating the findings of the focus groups.  Step 3 - Modelling process and outcome. The purpose of this step is to represent, in an understandable way, the interaction between the intervention components, mechanisms and outcomes. | Not Reported | We followed De Silva’s 3-step process (5) which supplemented and adapted the steps set out in the MRC guidance [28] for the ToC: Step 1 - identifying the evidence base; Step 2 - developing the theory through design intervention and creating realistic expectations, including in our case specific emphasis on SHIs; Step 3 - modeling process and outcome. It aims to guide the iterative development of a theory of change: combining outcomes, mechanisms and components.  Step 1 - identifying the evidence base. This step aims to identify and select data from the literture that could help stakeholders involved in phase 2 in designing the intervention theory. These data [...]  Step 2 - developing the theory. In keeping with theory-driven approaches, we put to-gether 4 expert focus groups and one user focus groupin [...] Finally, in the 3rd round of the e-Delphi procedure, we asked the professional group to validate the list of linkage mechanism(s)–BCTs adjusted.  Step 3 - Modelling process and outcome The purpose of this step is to represent, in an understandable way, the interaction between the intervention components, mechanisms and outcomes. An example of a ToCmap on peer counselling for maternal depression intervention has been presented by De Silva [10]. All the intervention theory elements were then modeled by the Xmind® software to present them in the form of a map. | Not reported |
| Barnhart et al. 2020 [18] | The ToC was developed primarily by evaluators and program implementers, but the prospective involvement of community members and frontline health providers can provide additional insight into the local context and increase community buy-in. | Not Reported | TOCs should include the complex intervention’s individual components, primary outcome, and any process outcomes hypothesized to be on the causal pathway between at least one intervention component and the primary outcome. Additionally, TOCs should contain information on contextual factors expected to modify the relationship between these variables. Although many researchers use the terms logic model and TOC interchangeably, TOCs necessarily include information about the assumed causal connections between variables while logic models often assume simplistic progressions between groups of variables, such as inputs, outputs, outcomes, and impacts (e.g.,) without making their causal assumptions explicit.  The theory of change (TOC) proposed in this paper was retrospectively developed following a review of the study materials and refined through discussion with members from the BetterBirth team. | Not Reported |
| Bonell et al. 2013 [21] | In stage 1, we searched for and constructed a descriptive map of all relevant theoretical and empirical references relating to the school envir-onment and health.  Stage 2 involved five separate syntheses, one on theory and fouron empirical research focused on the following health topics among students aged 4–18: aggressive behaviours, smoking, drinking and illicit drug use, sexual health, physical activity and diet, mental health, sun protection and accidental injury. | how systematic review methods might be employed to develop theories of change sufficient to inform ‘complex’ public-health interventions addressing multiple risks at the individual as well as community levels. | - In stage 1, we searched for and constructed a descriptive map of all relevant theoretical and empirical references relating to the school environment and health. - In stage 2, involved five separate syntheses, one on theory and four on empirical research focused on the following health topics among students aged 4–18: aggressive behaviours, smoking, drinking and illicit drug use, sexual health, physical activity and diet, mental health, sun protection and accidental injury. Stage 2 inclusion criteria for our review of theory were that reports: (1) presented or cited a named abstract, generalisable framework, developed by the authors or others, for how schools might influence student health; and (2) were written in English. Two reviewers first re-checked references that were described as concerning theory in the stage-one evidence map, to determine if they met these criteria. Second, reviewers screened all empirical reports included in our four syntheses of empirical evidence in stage two (n=79) to see if these cited theories that met the criteria above. Where the included report was not the original source of the theory, this was obtained. The original source was used to extract the theory’s name, constructs and pathways. We first narratively summarised each of our included theories, describing key concepts (which we italicise in our results section) and the inter-relationships between these. We then categorised each theory as ‘upstream’ (concerning schools’ organisation, teaching, discipline or pastoral care, or physical environment), ‘downstream’ (relating to students’ health-related cognitions or behaviour) or ‘medial’ (bridging between upstream and downstream pathways). In summarising theories, we identified key assumptions, commonalities and differences between theories, and drew out implications for school effects on health if these were not explicit. Informed by these narrative summaries and in particular by our categorisation of each individual theory as upstream, medial or downstream, we finally produced an integrated theory of how the school environment influences student health, charting complex pathways using a flow-diagram. | Not reported |
| Breuer et al. 2016 [23] | The ToCs were developed using workshops and working groups, document reviews, interviews and discussions, surveys, programme observation, literature reviews and existing conceptual frameworks or theory. The ToC development included consultations or interviews with the following stakeholders: programme staff, management, families, service users, experts and evaluators. Many used multiple methods, for example, Mookheriji and Lafond used immunisation programme theory and discussion with programme stakeholders, including immunisation experts, to develop a ToC of routine immunisation performance. They used a case study approach to evaluate immunisation performance and then refined the ToC based on the results of this evaluation and a stakeholder workshop. | Not reported | The ToCs were developed using workshops and working groups, document reviews, interviews and discussions, surveys, programme observation, literature reviews and existing conceptual frameworks or theory. The ToC development included consultations or interviews with the following stakeholders: programme staff, management, families, service users, experts and evaluators. Many used multiple methods, for example, Mookheriji and Lafond used immunisation programme theory and discussion with programme stakeholders, including immunisation experts, to develop a ToC of routine immunisation performance. They used a case study approach to evaluate immunisation performance and then refined the ToC based on the results of this evaluation and a stakeholder workshop. | Not reported |
| De Buck et al. 2018 [1] | A first draft of the ToC was developed by three of the team members, and with electronic input of the other team members. The draft ToC was based on different existing sources of information, including theoretical models, frameworks and systematic reviews. To identify these, (1) a literature review was performed to search for existing systematic reviews describing WASH promotion programmes and behaviour change in LMICs, and (2) methodological experts in programme implementation and qualitative research were consulted. This resulted in a list of resources that was used to create a first draft of the ToC.  The draft ToC was circulated electronically to the stakeholders, in preparation for a face-to-face meeting with the majority of the team members and stakeholders.  As a general remark, the stakeholders also noticed that it is relevant to them to know which elements of the ToC are based on evidence and which not. | Not reported | The methodology used to develop the ToC is schematically depicted in Figure 1, and includes the use of existing sources of information, followed by stakeholder consultation and external peer-review, which is described more in detail below. A ToC is composed of three major components: the intervention(s), the results and the factors influencing the different steps in the ToC (i.e. contextual factors and factors influencing implementation). The results are composed of outputs and outcomes, where outcome is defined here is as the effect that the intervention aims to achieve, and is composed of short-term outcomes (these outcomes that lead to actual WASH behaviour in case of our WASH example, called the ‘behavioural factors’), intermediate term outcomes (actual behaviour) and long-term outcomes (health outcomes). Contextual factors are characteristics of the environment which could influence the different links between intervention and programme outputs and outcomes, including socio-cultural, physical and personal factors. Factors influencing implementation are programme-related factors on the one hand, and factors leading to the behavioural factors (short-term outcomes) on the other hand. The latter are factors on the level of the implementer and the recipient (more detail below).  A first draft of the ToC was developed by three of the team members, and with electronic input of the other team members. The draft ToC was based on different existing sources of information, including theoretical models, frameworks and systematic reviews. To identify these, (1) a literature review was performed to search for existing systematic reviews describing WASH promotion programmes and behaviour change in LMICs, and (2) methodological experts in programme implementation and qualitative research were consulted. This resulted in a list of resources that was used to create a first draft of the ToC.  The draft ToC was circulated electronically to the stakeholders, in preparation for a face-to-face meeting with the majority of the team members and stakeholders (13 participants).  As a general remark, the stakeholders also noticed that it is relevant to them to know which elements of the ToC are based on evidence and which not. | Not reported |
| De Silva et al. 2014 [13] | Constructing a draft ToC framework is best done by project or programme stakeholders during a workshop, facilitated by someone who is experienced in using ToC. The other participants in the workshop do not need to have been exposed to ToC before, and do not need to know any of the terminology, as the facilitator can guide the group through the process of developing and refining the ToC. | Not reported | 1: Decide on the IMPACT in the real world you want to make and put this on the far right hand side of the wall (how communities will be  different because of what you do).  2: Brainstorm INTERMEDIATE OUTCOMES needed to achieve this impact and place them in a group on the far left hand side of the wall in no particular order. This is to encourage brainstorming of intermediate outcomesrather than being constrained by trying to think of intermediate outcomes as well as the order in which they come in the causal chain. As you start to get more intermediate outcomes, start placing them in the rough order they come in the causal chain. The process of listing intermediate outcomes and ordering them is an iterative process (Decide on the LONG TERM OUTCOME: This is the final outcome that the intervention is accountable for achieving; Decide on INTERMEDIATE OUTCOMES and determine the pathways that connect them: Work backwards through the logical steps),  3: As the Outcomes Framework is being populated, start adding the specific INTERVENTIONS that need to happen to move from one Outcome to the next (Map in the ToC the specific Intervention Components you need to do to achieve each Outcome )  4: At the same time, add any ASSUMPTIONS or RATIONALE to the links in the causal chain as they occur to the group (Rationale: Why do we think a given intermediate outcome will lead to (or is necessary to) reach the one above it? / Assumptions: Are there any major barriers to the intermediate outcome that need to be considered in our planning?).  5: Define INDICATORS of success for each of the intermediate outcomes:  For each intermediate outcome, choose at least one indicator to measure whether that intermediate outcome has been achieved. Then decide on how each indicator will be measured and by whom (evaluation methods). A critically important part of ToC is to decide HOW MUCH change is necessary in the intermediate outcome to move up the causal chain, or how much change is ‘good enough’. Pre-specifying the level of change needed to affect an intermediate outcome makes it easier to design the components of the intervention to affect that level of change.  Because intermediate outcomes are at different levels, indicators must also be measured at multiple levels, e.g. patient, community, stakeholders and care providers. This results in a more rounded evaluation with a wider range of indicators evaluated than is often the case. These indicators are in addition to the standard outcome measures of effectiveness (e.g. clinical and functioning patient level outcomes) or routine process indicators such as number of people trained adherence to medication or number of therapy sessions attended.  As well as WHAT to measure, ToC provides a rationale structure for WHEN to measure each intermediate outcome, as measurement points are determined by when the intended intermediate outcomes caused by the interventions specified in the ToC occur.  6: Be aware that interventions that take place later in the causal chain will be shaped by the intermediate outcomes of earlier interventions in the chain, so the ToC can evolve over time, and the exact nature of the later components of the intervention may not be known until later in the process (once the formative and piloting work has been done).  7: The key thing to focus on is MAPPING OUT THE INTERMEDIATE OUTCOME FRAMEWORK or causal pathway, and not get trapped into thinking about the specific intervention components that you think you will use, as this restricts your thinking as to what is needed to achieve the desired impact.  Assign a facilitator who is familiar with what ToC is and how to construct ToC map. This means that the rest of the group can get on with the brainstorming rather than needing to know about the specifics of how to construct a ToC, such as how to word intermediate outcomes appropriately. Decide the structure of the workshops based on the local context and the types of stakeholders. This will be very culturally dependent and how many workshops you have and who comes to them will very much depend on the local situation and the stakeholders you wish to include. For example, in some contexts you may need to hold a separate workshop for higher level government officials and a separate one for community health workers and service users, as the latter group would not feel comfortable contributing in the broader group. You may also decide to have a number of sequential workshops starting with a workshop to map the outcomes pathway followed up later with a second workshop to reflect on and refine this pathway, and to flesh it out with indicators and rationale. Ensure you have appropriate materials and space for developing the ToC as a group. It is easiest to construct the ToC map on a wall or similar surface that all participants can view easily. If you are using a wall, use large post-it notes of several coloursto denote the different component types so you can colour code the ToC map (for example all intermediate outcomes may be on green post-it notes, all interventions on smaller yellow post-it notes). Set the ground rules for the group based on the group dynamics. For example, if the group knows each other well a more informal approach may work better with everyone encouraged to move the post-it notes around and write on the notes. If the group is more formal or hierarchical then the facilitator can take the lead on this. | Not reported |
| Hartley et al. 2019 [36] | first, a literature review of effective interventions for parents in general was undertaken;  second, interviews with kinship carers were conducted in order to identify additional needs;  and third, stakeholder consultations were held to identify a specific population group. | Not reported | The 6SQuID framework is a pragmatic, evidence-based, co-produced, six-step framework for intervention development. It was recently developed in the field of public health, but it has wider applicability to other disciplines. It has been applied here to the area of kinship care, and therefore it is of relevance to social work. The framework comprises six steps, but can be categorised into two broad phases, and the paper will deal with each, in turn. The first phase focuses upon the nature of the problem (in this case, how to support the kinship-carers of teenage children), its causes, the factors which are open to modification, and the techniques which will effect beneficial changes. The second phase purports to clarify how the changes will be delivered, and it develops, tests and adapts the intervention programme at issue. In addition, some of the possible implications of the findings for public policy are considered.  The first three essential steps of the 6SQuID framework broadly allow for a theory of change to be generated which will inform the subsequent development, testing and adaptation of the CARE intervention.  3 first steps from 6SQuID:  1. Define and understand the problem and its causes Literature review, stakeholder consultations, interviews with kinship carers  In order to define the problem and its causes, and to identify which factors are malleable and can be modified as part of the intervention, three sources of data were collected and analysed: first, a literature review of effective interventions for parents in general was undertaken; second, interviews with kinship carers were conducted in order to identify additional needs; and third, stakeholder consultations were held to identify a specific population group.  2. Identify which factors can be modified  Based on the factors identified in Step 1, and from the previous literature review  3. Decide on the mechanisms of change  The final stage in the development of the first phase of the CARE programme was informed by Step 3 of the 6SQuID framework. This step involved identifying the techniques that will bring about change, and the intended outcomes deriving from them (Figure 2). This was developed based upon findings from Steps 1 and 2, and from discussions with the advisory group. | Not reported |
| Jamal et al. 2015 [37] | Our original theory of change drew predominantly upon sociological theory, focusing on system-level change. Before the earlier pilot trial, we described this initial theory of change using a diagrammatic logic model. | Stage 2: empirical process evaluation to refine CMO hypotheses. In this stage, which is ongoing, we will refine and augment the theory and hypotheses developed in stage 1 by drawing on empirical evidence. we are collecting data on intervention implementation, receipt, acceptability and normalisation (i.e. sustainability), as well as mechanisms and context.  Stage 3: testing mid-level CMO theories, In this stage, we will test hypotheses derived in stages 1and 2 via quantitative analyses of effect mediation (to examine mechanisms) and moderation (to examine contextual contingencies).  Combining process and outcome data enables us to develop empirically informed mid-range theory about school processes and how these may be modified by the intervention, and the extent to which LT may be transferable to a range of population and contexts. Causal mediation analysis helps to identify process or mediating variables that lie in the causal pathways between the treatment and the outcome. | Our first stage consisted of developing various a priori hypotheses about how intervention mechanisms might interact with context differentially to produce outcomes (CMO configurations). We did this at the start of the current phase III trial. The context-mechanism-outcome (CMO) hypotheses were informed by the findings of the prior pilot trial and an earlier feasibility study [15, 16], as well as by the sociological theory that informed the original intervention logic model and design [17] and empirical evidence regarding the processes of school effects on health [10, 18]. The second stage will involve refining or augmenting this limited list of a priori hypotheses prior to the collection of quantitative, follow-up data. This will be informed by the findings which will emerge from the process evaluation which is integral to the current phase III RCT. In the third stage, we will test our hypotheses using a combination of process and outcome data from the phase III trial: for example, to examine moderation and mediation.  Stage 1: pre-hypothesised theory of change and CMO configurations  + Pre-hypothesised intervention mechanisms (To examine empirically whether this is the case we will examine various mediation hypotheses suggested by this logic model and the intervention theory underlying it:)  + Pre-hypothesised contextual barriers and facilitators (priori hypotheses about how context might moderate intervention mechanisms and outcomes, informed by literature)  Stage 2: empirical process evaluation to refine CMO hypotheses :In this stage, which is ongoing, we will refine and augment the theory and hypotheses developed in stage 1 by drawing on empirical evidence.  + Data are being collected via:  ▪ diaries completed by intervention deliverers (trainers and external facilitators working in schools to establish and support action groups);  ▪ researcher observations;  ▪ interviews with school staff, students and intervention deliverers;  ▪ surveys that monitor satisfaction and implementation of core components of the intervention; and  ▪ in-depth case studies involving participant observation, focus groups and interviews in a selection of intervention schools.  Stage 3: testing mid-level CMO theories: In this stage, we will test hypotheses derived in stages 1 and 2 via quantitative analyses of effect mediation (to examine mechanisms) and moderation (to examine contextual contingencies). There are concerns among trialists that within trials multiple analyses can lead to false positive results [26]. However, our approach of grounding these in hypothesis testing minimises these risks and ensures transparency. | Stage 2: empirical process evaluation to refine CMO hypotheses. In this stage, which is ongoing, we will refine and augment the theory and hypotheses developed in stage 1 by drawing on empirical evidence.  Stage 3: testing mid-level CMO theories, In this stage, we will test hypotheses derived in stages 1and 2 via quantitative analyses of effect mediation (to examine mechanisms) and moderation (to examine contextual contingencies). |
| Lam et al. 2021 [24] | The development process varied across ToC studies, with methods ranging from participatory workshops which encouraged stakeholder participation, to evaluator-led approaches such as interviews. Another common approach was to draft an initial ToC by the evaluation team and then circulate the draft to program implementers and/or wider stakeholders for revisions. | Not reported | The development process varied across ToC studies (94 %; n = 59), with methods ranging from participatory workshops (n = 20) which encouraged stakeholder participation, to evaluator-led approaches such as interviews (n = 2). Another common approach was to draft an initial ToC by the evaluation team and then circulate the draft to program implementers and/or wider stakeholders for revisions (Johnson et al. 2015).  There were two main pathways through which “complexity-aware” ToCs were developed: (1) the study emphasized complex issues or systems (Douthwaite and Hoffecker 2017; Van Epp & Garside, 2019; Maru, Sparrow, Butler et al., 2018; Maru, Sparrow, Stirzaker et al., 2018), and (2) the study focused on evaluation challenges or information gaps while also emphasizing complexity (Douthwaite et al., 2017). Studies reported accounting for complexity through feedback loops (i.e. two-way arrows linking outcomes) and backward mapping (i.e. asking what are the necessary preconditions for outcomes to occur). The concept of emergence was considered in some studies through revisions made to the ToC as new information was gained during program planning or implementation. For example, in Apgar et al. (2017), the initial broad ToC for an aquatic agricultural systems program was revised after a planning workshop to further detail the ToC, leading to the integration of gender equity, nutrition, and climate change resilience needs. According to some authors, ToCs of complex programs were purposively simplified by presenting a variety of ToCs for actors, projects, or sites (i.e. nested ToCs). For instance, Douthwaite et al. (2013) developed a broad ToC at the program-level and several ToCs at the project-level.  Studies describing a program’s complexity, an evaluation challenge, or an information gap typically developed ToCs that appeared simple. Simple ToCs were often characterized by linearity, expressed through the use of uni-directional arrows between activities and outcomes (Pound 2015). In many studies, ToCs were described as following an “if/then” logic (i.e. if these activities are conducted, then these outcomes will be achieved) (Álvarez-Mingote et al., 2020; Baker et al., 2013; Levay et al., 2018), suggesting a stepwise pathway toward food security outcomes. | Not reported |
| Mayne; Johnson, 2015 [38] | Often one develops a ToC based on both stakeholder views and relevant research findings. | Not reported | Can start with a blank page or with a straw impact pathway/theory of change In a participatory manner, one can start with a blank page and a facilitator who proceeds to tease out the way participants (stakeholders) imagine that the intervention is to work and the implicit assumptions behind their thinking. By continually asking ‘why?’ and challenging the ideas put forth, an IP/ToC will emerge. A key advantage in this approach is that there will likely be considerable buy-in the resulting IP/ToC. It may, however, take some time for an agreed IP/ToC to emerge. Alternatively, a few people can draw up a seemingly reasonable IP/ToC and offer it as a starting point for debate and discussion. Stakeholders typically have strong views about how they think the intervention is to work, so a lively debate usually follows. This approach usually results in a more efficient process but may not get the same level of buy-in as quickly as the blank page approach. It has another advantage though. It may be easier to bring relevant prior research and evaluation findings to bear to buttress the IP/ToC. Include explicitly or implicitly all the impact pathway/theory of change elements The suggestion here is that in most interventions each of the ToC components (reach, capacity changes, behavioral change, direct benefits, livelihood change) can be, and should be, identified, as building blocks of a ToC. Theory of change should be seen as a process, evolving over time as more insight is gained Both Vogel (2012a) and Barnett and Gregorowski (2013) stress the importance of seeing and using ToCs as a tool throughout the life of an intervention. The ToC can be a useful tool for reflecting on the implementation of the intervention. Of course, one might conclude that results are not occurring as expected at all and decide to completely rethink the ToC or indeed stop the intervention. Are based on prior research and stakeholder views A good IP/ToC is not just the agreed views and beliefs of stakeholders. It should also be as much as possible evidence-based, using research and evaluation findings on previous similar interventions to identify likely linkages, rationale and causal link assumptions and risks (Stern et al., 2012; Vaessen and Leeuw, 2009). All of the A4NH interventions and the corresponding ToCs developed are based on previous research and on evidence from pilot interventions which should be used to inform current efforts. Since ToCs are based on a combination of prior social science research, experience and on stakeholder views, a number of possible ToCs for a given intervention can be generated. Hence the need to see a ToC as a model hypothesis at a point in time which is developed to be tested: • One might want to test a funder’s ToC about how things are supposed to work, or a hypothesis about underlying social processes. • Often one develops a ToC based on both stakeholder views and relevant research findings. • There may be cases where there are quite different views on how an intervention is to work. One may then need more than one ToC and each gets challenged and tested (Hansen and Vedung, 2010). | Not reported |
| Meiksin et al. 2021 [29] | Authors mentioned many scientific theories that informed the intervention ToC, such as information-motivation-behavioural skills (IMB) model and social cognitive theory. | Not reported | We undertook synthesis of author narratives describingtheories of how interventions were intended to generateoutcomes. We aimed first to summarise theories of change for specific interventions and then to examine whether there were one or more overarching theories of change relevant across different interventions. Theory synthesis commonly uses a meta-ethnographic approach,originally developed to synthesise findings across mul-tiple qualitative studies, and now applied to theory synthesis. As originally applied to qualitative research,meta-ethnographic methods draw on primary constructs(verbatim qualitative data presented in reports of pri-mary research) and secondary constructs (authorinterpretations of data presented in primary research) todevelop tertiary constructs (reviewer interpretations pre-sented in syntheses). Applied to theory synthesis, suchmethods draw solely on primary constructs (author de-scriptions of theory of change).We initially planned to undertake line-by-line codingof theory reports in order to identify narrative themeswithin, and common themes across, intervention theor-ies of change as we have done in previous theory synthe-ses. In the first stage of our analysis tworeviewers piloted this approach, using data extractedfrom the two highest-quality studies of similar interven-tions. We applied line-by-line codes, beginningwith in vivo codes which closely reflected the wordsused in the theory reports. We then grouped and orga-nised codes, applying axial codes reflecting higher-orderthemes and met to compare and contrast the resultingcoding. Because this approach did not readily capturethe complex and well-described interrelationships be-tween theoretical constructs present in the reports, we in-stead decided to develop a novel diagrammatic approachto theory synthesis. This methodological innovationallowed us to summarise the components of each inter-vention’s theory of change and the explicit and/or impliedcausal relationships between them, drawing on text anddiagrams present in the studies. Summarising these dia-grammatically also facilitated comparison and synthesis ofthese components and relationships across included the-ories of change. Specifically, we drew diagrams of theoriesof change based on author text and diagrams, first foreach intervention and then for overarching theories ofchange, which applied across multiple interventions. Likethe approach we had initially planned, this novel methodof theory synthesis was a form of qualitative synthesis, butone that aimed to describe theories of change primarily interms of constructs, inter-connections and interactions ra-ther than as narrative themes. Like conventional thematicanalysis, it involved an initial stage of‘in vivo’coding ofauthor descriptions to identify theories of change for eachintervention (but expressed diagrammatically rather thanas a set of narrative themes), followed by a stage of‘axial’coding to explore inter-connections between in vivo cod-ing, identifying similarities and differences across interven-tions to develop overarching theories of change (againexpressed diagrammatically).Where more than one publication reported on thesame intervention, reviewers drew on the theory ofchange descriptions from all relevant reports to informthe diagram. The two reviewers then met to comparethe two diagrams for each intervention and reconcilediscrepancies through discussion. Drawing on thestrengths of each, we developed an overall diagram of eachintervention’s theory of change, which included interven-tion components, mediators and moderators (where discussed by authors) and intended outcomes. Where au-thor descriptions implied but did not explicitly state inter-relationships between components of the theory ofchange, reviewers made inferences and noted where thediagrams were in part based on such inferences.Finding that the theory of change approaches under-pinning the interventions were not patterned by targetedoutcomes, we took an inductive approach, grouping dia-grams of theories of change that shared important con-structs. Then, using reciprocal translation (to identifyand describe similar concepts occurring across theoriesof change underpinning different interventions), refuta-tional synthesis (to identify contradictory or opposingconcepts occurring across theories of change) and lineof argument (to synthesise distinct elements occurringacross theories of change that form part of a broaderwhole) approaches from meta-ethnography [57], each re-viewer independently analysed the diagrams within eachgrouping. They did this by systematically examining theconstructs and the relationships between constructs pre-sented in each intervention-specific diagram and byexamining whether they recurred, appeared only once orconflicted with those depicted in other intervention-specific diagrams within the grouping. Based on theiranalyses, each reviewer then independently drafted onesynthesised diagram for each grouping of similar inter-vention theories of change.We documented each stage of this process, notingwhere theory of change components or relationships be-tween these components differed between individual dia-grams within the grouping; the approach taken tosynthesise these components (i.e. reciprocal translation,refutational synthesis, line of argument or the exclusionof a theory of change component); and the resulting de-cision for the synthesised theory of change diagram. Thetwo reviewers then met to compare their respective syn-thesised diagrams for each grouping, reconciling discrep-ancies and drawing on the strengths of each to develop asingle synthesised theory of change diagram for eachtheory of change grouping. To demonstrate this process,Additional file2presents the theory of change diagramsfor each individual intervention in one grouping and theresulting diagram of the synthesised theory of change forthat grouping. Each synthesised theory of change wasgiven a descriptive title inductively drawing on the cen-tral approaches of the theories of change synthesised.In this application of meta-ethnographic methods tothe synthesis of theories of change, our first-order con-structs were the theory of change information describedin theory reports and represented in data extractionforms; our second-order constructs (analogous toin vivo codes) were the reviewers’interpretations ofthese concepts, represented in the intervention-specifictheory of change diagrams and our third-orderconstructs (analogous to axial codes) were the higher-order interpretations, represented by the diagrams ofthe synthesised theories of change developed for eachinductive grouping.  Whilst we have previously conducted reviews synthe-sising intervention theories of change using line-by-linecoding of descriptive text, we found that this ap-proach did not capture the often precisely described andcomplex inter-relationships between theoretical con-structs presented in the body of literature for this review.Theories of change included in our past reviews, whichaddressed the integration of health and academic educa-tion and positive youth development interventions, were relatively simple and either not significantlyinformed by existing scientific theories or informedby theories that are not typically portrayed visually.In contrast, theories of change underpinning the e-health interventions included in this review were morecomplex, more explicitly theorised and largely drew onexisting scientific theories which are typically conceptua-lised in terms of diagrams indicating relationships be-tween their theoretical constructs, with constructswidely recognised and understood and therefore not al-ways discussed at length. Whilst thematic analysis is agood way of rendering explicit what is implicit, it is lessappropriate where the literature itself is more explicit.We therefore developed a novel method of theory ofchange synthesis in which we created diagrammaticsummative logic models of intervention theories ofchange. By inductively grouping these models accordingto their core constructs and using meta-ethnographicapproaches, we identified three emergent theoretical ap-proaches underpinning the included interventions andwe created synthesised models of each approach. Wehave thus synthesised theories of change underlyinginterventions with similar approaches. These summariseand integrate existing theories of change rather thanproviding a novel overarching theory of change for suchinterventions. | Not reported |
| Osterman et al. 2021 [39] | The hypotheses and assumptions reflected in the TOC were informed by existing health information and data use frameworks, as well as systematic reviews on topics related to health information system strengthening and evidence-informed decision-making.  From this literature, we identified six barriers to data use (demand, access and availability, quality, skills, structure and process, and communication) and three behavioral drivers (capability, motivation, and opportunity), which are represented in the TOC as mechanisms of data use interventions. | Not reported | The hypotheses and assumptions reflected in the TOC were informed by existing health information and data use frameworks, as well as systematic reviews on topics related to health information system strengthening and evidence-informed decision-making [11,12,13,14,15,16].  From this literature, we identified six barriers to data use (demand, access and availability, quality, skills, structure and process, and communication) and three behavioral drivers (capability, motivation, and opportunity), which are represented in the TOC as mechanisms of data use interventions [11,12,13,14]. We hypothesize that to be effective, any intervention must address one or more of these mechanisms. Likewise, we expect that interventions addressing these mechanisms will lead to intermediate outcomes including data quality and availability, analysis, synthesis, and discussion of data, which we posit are also necessary precursors to data use. The data use actions represent our outcome of interest in this review; they specify where data are used, by whom, and for what purpose. | Not reported |
| Rippon et al. 2017 [40] | Not reported | The comments in our field discussions suggest to us a need to develop a further element in a ToC for asset based approaches. | Think Piece meetings with stakeholders  We saw the ToC approach as a means to articulate the key stages for adopting asset based approaches in local systems. The key stages of development include the mapping of the logical sequence of ‘event’ or actions in the overall ‘programme’ underpinned by critical questioning and scrutiny of the contextual conditions that will and are influencing the action. This, includes thinking about the contributions of key stakeholders, resources and the assumptions and interpretations being made as to how and why the sequence of change will come about.In practice, in progressing through these stages of development and delivery of the ToC it is important to sense check the progress with relevant evidence (both qualitative and quantitative) to generate deeper insight and firm foundations for the operating ToC model.ibid p6.The stages of this ToC model are not necessarily linear and may be ordered to suit the context; when adopting the ToC local agents should look to create a process wherein each stage can have a set of descriptors that illustrate actions, purpose and impact. For each stage of the ToC, there will be micro detail to show context, assumptions, resource inputs, intermediate outputs and outcomes.In summary, the revised ToC includes an orientation phase, setting out purposeful intent and a rationale for action; this includes: – Adopting participatory approaches that foster and strengthen engagement and involvement of people – creating dialogue, inclusive opportunities to plan and decide on actions required. – Agreeing and being clear on the purpose of planned actions – e.g. to develop a health assets model.– Undertaking a review/mapping of resources, including knowledge, skills, relationships etc. that will boost adoption of purposeful asset based approaches.– Agreeing at what level asset based approaches are being adopted – see: Appendix: Diagram One.The subsequent and complementary phases of the ToC include reframing current and established activity: – Reframing existing relationships and the use of resources toward the purpose agreed in the orientation phase.– Introducing asset based approaches to reframe dialogue, planning and action with those already engaged.Where action has been taken to Map Assets the perspectives and knowledge gained from this can be reframed and applied to the orientation phase to:– Better understand current health assets in place.– Identify the location of assets – in neighbourhoods, communities, organisations etc.– Build on the range of assets available to support action.These actions can then be part of the mobilisation phase of the ToC, using existing assets to support the agreed direction of development. | The comments in our field discussions suggest to us a need to develop a further element in a ToC for asset based approaches. |
| Sapkota et al. 2019 [14] | The process of developing a ToC was initiated with an identification of long-term goals of the included interventions. Subsequently, the reviewers worked backwards to develop a pathway of change illustrating the cause-effect relations between the activities and the intended outcomes. Each output was interconnected; in that they influenced and supported the outcomes and facilitated the achievement of desired impacts. Assumptions were articulated to explain the linkages between the activities and outcomes and were supported by existing theories and findings from the included studies. | Not reported | The process of developing a ToC was initiated with an identification of long-term goals of the included interventions. Subsequently, the reviewers worked backwards to develop a pathway of change illustrating the cause-effect relations between the activities and the intended outcomes. Each output was interconnected; in that they influenced and supported the outcomes and facilitated the achievement of desired impacts. Assumptions were articulated to explain the linkages between the activities and outcomes and were supported by existing theories and findings from the included studies.  1. Narrative theory of change (ToC) for a DV intervention (A theory of change for interventions addressing DV among pregnant women in LMICs Figure4).  - Context  The foundation of the ToC begins with a core problem that DV during pregnancy contributes to a larger proportion of maternal and neonatal morbidities and mortalities in LMICs.  - Activities  A number of intervention strategies or activities contribute directly or indirectly to overcome these barriers, which are described below in brief. | Not reported |
| Tancred et al. 2018 [41] | The evidence for the development of ToC came through educational strategies  1. 4Rs (Reading, Writing, Respect, and Resolution)  2. Positive Action (PA)  3. Promoting Alternative Thinking Strategies (PATHS)  4. Raising Healthy Children  5. Roots of Empathy  6. The Gatehouse Project  7. Youth Matters | Not reported | ToC was developed in different educational activities:  1. 4Rs (Reading, Writing, Respect, and Resolution)  2. Positive Action (PA)  3. Promoting Alternative Thinking Strategies (PATHS)  4. Raising Healthy Children  5. Roots of Empathy  6. The Gatehouse Project  7. Youth Matters | Not reported |
| Tirman et al. 2021 [42] | 6SQuID model  Step 3: Developing a theory of change involved developing intervention activities to target each modifiable factor leading to sedentary behaviour. This step details how each modifiable factor identified in step 2 could be addressed at each level of the contact centre by designing specific activities informed by the focus groups, literature review and workshop data. | Not reported | 3. Defining the theory of change:  Development of a logic model for the theory of change  Workshop with contact centre staff  Step 3: defining the theory of change: understanding the change mechanisms  Following the focus groups, a workshop was held at the Ipsos MORI contact centre where all staff members were invited to drop in, try out equipment, participate in mindfulness activities like colouring, jigsaw and Lego, and then tell us about their preferences. Thirty-six staff attended the workshop. The workshop activities were chosen by the research team based on feedback from the focus groups, and whether the activities were associated with one of the four levels from the socioecological framework leading to sedentary behaviour (individual, social/community, environmental, and organisational). The workshop included equipment such as standing desk risers, a treadmill, a desk bike, exercise bands, stepper machines, exercise bands and more. Staff at the workshop rated pieces of equipment and individual and social activities they were interested in through a prioritisation exercise by placing sticky dots next to names of activities on flipcharts. The research team provided ideas to support the exercise, suggesting individual activities such as goal setting, taking the stairs, and walking or cycling to work. They also suggested social activities such as group competitions, group exercise classes, and other social events. The research team used the findings from the prioritisation activity to inform the final decision of which activities were to be implemented. This decision also took into account the context of the contact centre (e.g. centre layout, work-time flexibility, budget and resources available) and the requirement to follow the programme theory, implementing activities that target sedentary behaviour at each level of the centre (individual, social/community, environmental, and organisational). Lastly, a logic model to illustrate the theory of change was developed. The intervention would focus on this change theory, rather than being prescriptive about activities that catalyse change. | Not reported |
| Yearwood, 2018 [43] | To identify long-term goals and map preconditions, we searched for published and gray literature that investigated application of evidence in decision-making in the Caribbean. | Not reported | Using the approach suggested by the World Bank (25) and Morra and Rist (26), we constructed the ToC using the following steps: i) identification of long-term goals; ii) backward mapping and connecting the preconditions to achieve goals; and iii) identifying interventions to generate desired change. Prior to goal determination, we also included a problem identification step, to ensure that the model's expected results were directly linked to the problems faced by Caribbean policymakers. | Not reported |

Source: authors' elaboration.
