# Supplementary material for: Integration of evidence into Theory of Change frameworks in the healthcare sector: A rapid systematic review
Source: PLoS One. 2023 Mar 9;18(3):e0282808. doi: 10.1371/journal.pone.0282808 (PMC9997872; doi:10.1371/journal.pone.0282808)
Supplement: Attachment — Submitted filename: Response to Reviewers.docx [file pone.0282808.s008.docx]

# Response letter to reviewers

Dear Reviewers,

We appreciate your time reviewing our paper and providing valuable feedback. Your careful reading and insightful comments led to improvements in the current version. The authors have carefully considered the comments and tried our best to address all of them. We hope the new manuscript meets your standards. The authors welcome further constructive comments, if necessary. Below we provide the point-by-point responses. All modifications in the manuscript have been highlighted with track changes.

Sincerely,

The Authors

# Authors replies to reviewers responses

**1. Is the manuscript technically sound, and do the data support the conclusions?**

*The manuscript must describe a technically sound piece of scientific research with data that supports the conclusions. Experiments must have been conducted rigorously, with appropriate controls, replication, and sample sizes. The conclusions must be drawn appropriately based on the data presented.*

Reviewer #1: Yes

Reviewer #2: Yes

**Authors:** No further changes to the manuscript were made in this regard.

**2. Has the statistical analysis been performed appropriately and rigorously?**

Reviewer #1: N/A

Reviewer #2: N/A

**Authors:** No further changes to the manuscript were made in this regard.

**3. Have the authors made all data underlying the findings in their manuscript fully available?**

*The* [*PLOS Data policy*](http://www.plosone.org/static/policies.action#sharing) *requires authors to make all data underlying the findings described in their manuscript fully available without restriction, with rare exception (please refer to the Data Availability Statement in the manuscript PDF file). The data should be provided as part of the manuscript or its supporting information, or deposited to a public repository. For example, in addition to summary statistics, the data points behind means, medians and variance measures should be available. If there are restrictions on publicly sharing data—e.g. participant privacy or use of data from a third party—those must be specified.*

Reviewer #1: Yes

Reviewer #2: Yes

**Authors:** No further changes to the manuscript were made in this regard.

**4. Is the manuscript presented in an intelligible fashion and written in standard English?**

*PLOS ONE does not copyedit accepted manuscripts, so the language in submitted articles must be clear, correct, and unambiguous. Any typographical or grammatical errors should be corrected at revision, so please note any specific errors here.*

Reviewer #1: Yes

Reviewer #2: Yes

**Authors:** No major changes to the manuscript were made in this regard, although in addressing some of the comments mentioned below we have edited some language to improve clarity.

**5. Review Comments to the Author**

*Please use the space provided to explain your answers to the questions above. You may also include additional comments for the author, including concerns about dual publication, research ethics, or publication ethics. (Please upload your review as an attachment if it exceeds 20,000 characters)*

**Reviewer #1:**

This review is clearly written, well organised and reported the strategies using research evidence when developing or adapting the 'Theory of Change' in healthcare context. This review also proposed the stages for 'Theory of Change development. A good dept and explanation in the results and discussion sections by the authors.

**Authors:** We appreciate the positive feedback. No further changes to the manuscript were made in this regard.

**Reviewer #2:**

**1. There is a reference to the "Theory of Change" on line 52 (the first line of the article). Prior to that, authors should define and explain what "the theory of change" is, in simple and easy words. Describe what you mean by "change" in this context, how the "theory of change" came to be, and give a background on the theory of change.**

**Authors:** We included the following explanation, in the first paragraph of the text, line 52-56:

“A Theory of Change (ToC) comprises a sequence of causal steps that describe how an intervention is expected to produce certain outputs and outcomes. These steps often include what inputs will be used, which activities will be conducted, what outputs will be produced, and, finally, what outcomes are expected to be achieved through this process, i.e, what “change” is expected to happen [1, 2].”

**2. Authors should explain how the ToCs were developed.**

**Authors:** We added some historical background to the concept of ToC, starting from line 62:

“The concept of ToC was preceded, during the 50s and 60s, by an approach called Program Theory, which proposed a way of understanding and planning interventions with focus on the roles of context, input, processes, and products for intervention design [5]. Over time, increasingly more attention was given to explaining how an intervention was expected to work in terms of its underlying causal links [6]. In 1995, The Roundtable on Comprehensive Community Development, supported by the Aspen Institute [7], advocated for the importance of making it clear for stakeholders the assumptions of how change is to be achieved by a program, popularizing the term Theory of Change, described as “a theory of how and why an initiative works” [8].”

**3. It might be helpful to define some of these lesser-known terms like ToC, logframes and logic model.**

**Authors:** We modified the paragraph where the terms Logframes and Logical Models are mentioned, as follows (line 70-83):

“The concept of ToC is related to, and often overlaps with, several other tools and frameworks. A Logical Model, for instance, is defined as a graphical representation of a program theory, showing the relevant causal connections up to the results [5]. A Program Theory is an explicit theory of how an intervention is expected to achieve its goals [5]. An Action Theory, on the other hand, describes the pathways for developing an intervention. Finally, a Logical Framework, or Logframe, is a method for planning and evaluation, usually specifying inputs, activities, outputs, outcomes, and impacts in a matrix, and possibly also including indicators and assumptions [9,10]. Logframes are considered less flexible than ToC diagrams, and more limited in representing multiple, interacting, and nonlinear causal pathways. Importantly, ToCs should make assumptions and contextual factors explicit to explain the causal steps, i.e. why and how an intervention is expected to lead to the anticipated changes [9]. Furthermore, a core feature that distinguishes ToC from other similar approaches is the emphasis on it being a living process, constantly updated based on lessons learned [11,12].”

**4. In line 97 change the word "ex-post" or explain it (based on analysis of past performance).**

**Authors:** We replaced the term “ex-post” with “after an intervention has been implemented” (line 130).

**5. There was no explanation provided by authors regarding the word "health sector" before objectives. Authors should provide information about how ToC is used in the health sector. If there is no literature available or none of the recent studies has systematically investigated the use of evidence to inform ToCs in the health sector, then how would you conduct a systematic review in the health sector?**

**Authors:** We have added the following details regarding how ToC is currently used in the health sector (line 136-161):

“ToC has been widely used in the health sector for planning, depicting, implementing, and evaluating interventions [11,12,23,24]. For instance, Barnhart et al. (2020) [18] developed a ToC based on the experience of three different implementation phases of a maternal and child health program, the Better Birth program [18]. Barriers, lessons learned and ways of applying these lessons were identified, and later integrated into a renewed ToC. In another example, Aggarwal (2021) [19] reviewed the literature for potential interventions and delivery mechanisms for reducing the self-harm of individuals in countries of low and middle income [19]. Based on the findings of the review, the authors propose a ToC for a model of delivery of psychosocial interventions. Furthermore, in the field of Implementation Science, ToC has been a recurring choice to describe models, plan solutions to complex problems and evaluate programs in the health area, as described by Breuer et al. (2016) [23]. Recent reviews suggest there is a growing number of published evaluations in the health area that use ToC, as identified by Breuer et al. (2016) [23] and Lam et al. (2021) [24]. However, both reviews highlight that studies, overall, lack an appropriate level of detail regarding how the ToCs were developed and used.”

**6. The authors did not explain the rapid review.**

**Authors:** We included a more detailed explanation about our choice to conduct a rapid review (line 242-254):

“This project applied a rapid review methodology. In a rapid review, evidence is synthesized and its validity is assessed using an abbreviated systematic review method, so to identify results in a shorter timeframe. Regardless of the abbreviated approach, this endeavor followed the principles of the scientific method and the key principles of evidence synthesis, including rigor, transparency, and reproducibility. This rapid review was conducted and reported following the process outlined by Tricco et al. (2017) [27]. We used a rapid review approach to be able to provide high quality evidence for decision making, in a timely way. We used a rapid review approach to be able to provide high quality evidence for decision making, in a timely way. Given the time and budget limitations of this project, some adaptations were made to adjust to the needs of the rapid review design. These adaptations are described in the section “Shortcuts taken for the rapid response” below.”

**7. In line no 148, the authors stated that "This rapid review adds to the existing literature" as "it provides an up-to-date and comprehensive review of the existing methods". Authors should discuss those existing methods first and then justify this statement.**

**Authors:** We edited the excerpt between lines 194 and 197 to meet the suggestion:

“Most of the existing literature on the topic focuses on the development of a ToC but only gives brief descriptions of the methods applied to incorporate evidence into this process. These methods include, for instance, structured stakeholder consultation and literature searches.”

**8. In line no 148, "we propose a sequence of 7 stages relevant to the process of evidence incorporation into ToCs". How you came up with these 7 stages. The authors should provide a description of the 7 stage process and how it came to be.**

**Authors:** We edited the excerpt in line 219 to indicate where this information is detailed in the text:

“The process for developing these 7 stages is described in the “Data synthesis” section below.”

**9. In line no 172, "Given the above-mentioned constraints, some adaptations were made to adjust to the needs" kindly provide the line number where the authors mentioned those constraints above.**

**Authors:** We have edited the excerpt in line 252 to include the constraints:

“Given the time and budget limitations of this project, some adaptations were made to adjust to the needs of the rapid review design. These adaptations are described in the section “Shortcuts taken for the rapid response” below.”

**10. In line no 196, please indicate the starting and ending date of articles searched. Or did authors search only in a day?**

**Authors:** Yes, the full search was conducted on this date.

**11. In line 199, the authors mention a topic expert. Who is that expert? What are the criteria for becoming an expert, and why only one expert? Also why additional search for gray literature was not conducted?**

**Authors:** We replaced the expression "topic expert" by an explanation about the source of the list of references. Regarding the lack of additional searches for gray literature, we added a phrase indicating this was due to time constraints (line 282-283).

“A list of references obtained from a WHO researcher that had been previously investigating the topic was also screened. Due to time constraints, no additional search for gray literature was conducted.”

**12. In lines no 297-299, the authors mentioned studies with high-income countries, low and middle-income countries and high-income economies "Seven studies were carried out in countries 298 that, according to The World Bank (2021) [37], are classified as high-income economies (HIE) 299 [10,12,17,27,29,30,35], three in Lower-middle-income economies (LMIE) [5,10,27]". In this sentence reference number 10 is mentioned in both high-income economies and lower-medium-income economies. Aggarwal, S., Patton, G., Berk, M. & Patel (reference 10) is only represented in LMIE. Also, check the reference no 27.**

**Authors:** We changed the excerpt between lines 398 and 401 to present separately studies that focused on both HIE and LMIE:

“Regarding the countries conducting the studies, five studies were carried out in countries that, according to The World Bank (2021) [43], are classified as high-income economies (HIE) [21,23,35,36,41], one in Lower-middle-income economies (LMIE) [14], and one in Upper-middle-income economies (UMIE) [42]. Two studies included both HIE and LMIE [18,19]”

**13. Kindly check reference no 30 in line no 309. In my opinion, this reference does not match the criteria.**

**Authors:** We added a new theme to better represent reference 30 (line 410): “realist RCTs [30]”.
