# Supplementary material for: Integration of evidence into Theory of Change frameworks in the healthcare sector: A rapid systematic review
Source: PLoS One. 2023 Mar 9;18(3):e0282808. doi: 10.1371/journal.pone.0282808 (PMC9997872; doi:10.1371/journal.pone.0282808)
Supplement: Attachment — Submitted filename: Response to Reviewers.docx [file pone.0282808.s009.docx]

# Response letter to reviewers

Dear Reviewer,

We appreciate your time reviewing our paper and providing valuable feedback. As before, we have carefully considered the comments and tried our best to address all of them. We hope the new manuscript meets your standards. The authors welcome further constructive comments, if necessary. Below, we provide the point-by-point responses.

Sincerely,

The Authors.

# Authors replies to reviewers comments

**[Reviewer #2]**

***1.*** *In line 70, "The concept of ToC is related to, and often overlaps with, several other tools and frameworks." Authors should mention the other tools and frameworks' names first with this sentence and then explain them.*

**Authors:** As requested, we added the names of the frameworks to the sentence. Included between lines 70-71:

The concept of ToC is related to, and often overlaps with, several other tools and frameworks, such as Logical Model, Program Theory, Action Theory or Logical Framework.

***2.*** *The authors mentioned different line numbers in the "Authors' replies to reviewers' responses" and the corrections done on different lines in the article. This confusion made the review process difficult.*

**Authors:** We apologize for the inconvenience. The formatting of the text apparently changed as we prepared the documents for the submission process. We tried to prevent the same problem for this new submission.

***3.*** *In line 200, kindly explain the "rapid review" with references.*

**Authors**: We understood this comment as the reviewer requesting us to define Rapid Review. We’ve added a definition between lines 213-215:

Rapid reviews can be defined as a type of literature review in which “the steps of the systematic review are streamlined or accelerated to produce evidence in a shortened timeframe." (p. XIII) [27].

***4.*** *In lines 205 and 206, authors repeated the same sentence: "We used a rapid review approach to be able to provide high quality evidence for decision making, in a timely way."*

**Authors:** The duplicate sentence has been deleted.

***5.*** *In the previous comment, "comment no. 7," the authors were asked to discuss those existing methods first and then justify this statement. However, the authors only stated that "the majority of the existing literature on the topic focuses on the development of a ToC but only provides brief descriptions of the methods used to incorporate evidence into this process."The authors did not explain the existing methods.*

**Authors:** The section between lines 157 and 180 was edited, so that the presentation of the existing methods comes before the general statement about them:

Two literature reviews have previously investigated the methods used to incorporate evidence into ToCs in the health sector. In a systematic review, Breuer et al. (2016) [23] investigated how ToCs are used to design and evaluate public health interventions. The authors identified the following methods used to incorporate evidence into ToC frameworks: “workshops and working groups, document reviews, interviews and discussions, surveys, program observation, literature reviews, and existing conceptual frameworks or theory, [...] consultations or interviews [with stakeholders]” (p.4). Moreover, in a scoping review, Lam et al. (2021) [24] investigated how ToCs are used in food security contexts. The authors find that the methods applied for using evidence in ToCs were primarily participatory approaches, such as workshops or interviews, i. e., favoring tacit knowledge. They also report that most studies do not provide details on how the ToCs were developed, which is “consistent with the sentiments in the literature, which highlighted the limited guidance, training, and agreement on how to develop a ToC” (p.7).

In this review, we expand the existing literature by focusing on how to systematically use research evidence to inform the development of ToCs in the health sector. Consistent with the previous reviews, our findings below show that, almost none of the recent studies have systematically investigated the use of evidence to inform ToCs in the health sector. Furthermore, we did not encounter any study that provides a comprehensive and rigorous account on what methods can or should be used to incorporate evidence into ToCs. Most of the primary studies that address how a ToC was developed only give brief descriptions of the methods applied to incorporate evidence into this process. These methods include, for instance, structured stakeholder consultation, literature searches and the use of institutional data, which are applied as sources of information to draft or improve ToCs. However, each study seemed to choose specific versions of these methods (e.g. stakeholder consultations) without a clear rationale for why this choice was made over the alternatives (e.g. a literature review).

***6.*** *In the previous comment, "comment no. 8," the authors were asked, "How did you come up with these 7 stages?" The authors' reply is not satisfactory. Authors should explain the details of 7 stages before applying them.*

**Authors:** We understand the reviewer wanted more details on how the 7 stages were developed. Since this is a question regarding our methods, between lines 192 to 196 we added a short description of the process, as well as between lines 308-319 and 324-325, in the section “Data synthesis”, we added further details about this process:

Pages 192-196:

These 7 stages were developed through a qualitative analysis of the data extracted from the included studies (further details provided in the “Data Synthesis” section below). Each stage corresponds to a specific set of objectives. The stages are: 1) "Define the problem", 2) "Define expected outcomes", 3) "Define interventions", 4) "Define change mechanisms", 5) "Model ToC", 6) "Validate ToC" and 7) "Revise ToC".

Lines 308-319:

The basis for this process was the extracted data regarding the steps for developing and revising a ToC and the strategies for evidence use (see S6 Appendix for the relevant extracted data). Through an inductive qualitative data analysis process, we created a typology of stages and coded the extracted information accordingly. When grouping the steps based on content similarity, we realized they could be classified with labels similar to the phases often included in the “policy cycle”, such as “Policy Formulation”, “Implementation” and “Evaluation” (p.33) [34]. Thus we regrouped the data following this framework, adapting some of these phases as labels (i.e. "Define the problem", "Define interventions"), and creating new ones when necessary (i.e. "Define change mechanisms", "Model ToC", "Validate ToC", "Revise ToC") based on our content similarity analysis. Here, we present these labels as stages for the development of ToCs. The different stages reflect the objectives and functions of each step, as shown in section “Stages for the development of a ToC” below and in **S3 Appendix**.

Lines 324-325:

Within each stage, we grouped the included methods according to the specific actions that were conducted.
